# Health care management adequacy among French persons with severe profound intellectual and multiple disabilities: a longitudinal study

**DOI:** 10.1186/s12913-024-10552-9

**Published:** 2024-01-18

**Authors:** Karine Baumstarck, Ilyes Hamouda, Marie-Anastasie Aim, Any Beltran Anzola, Sherezad Khaldi-Cherif, Agnès Felce, Kim Maincent, Katia Lind, Pascal Auquier, Thierry Billette de Villemeur, Marie-Christine Rousseau, and the EVAL-P. L. H. Group, Narjess Boutalbi, Lionel Dany, Ponha Heng, Patrick Julien, Isabelle Kemlin, Stéphane Lenormand, Stéphane Pietra, Julie Roger, Maria Valkov, Daniel Willocq

**Affiliations:** 1 EA 3279, CEReSS - Research Centre On Health Services and Quality of Life, Aix Marseille University, 27 Boulevard Jean-Moulin, 13385 Marseille, France; 2 Epidemiology and Health Economy Department, Assistance Publique Hôpitaux de Marseille, 27, Boulevard Jean-Moulin, 13385 Marseille, France; 3 UR 849, LPS - Social Psychology Laboratory, Aix-Marseille University, 29 Av. Robert Schuman, 13621 Aix-en-Provence, France; 4 General Union Health Insurance Fund (Union Générale Caisse Assurance Maladie, UGECAM), 26-50 Avenue du Professeur-André-Lemierre, 75986 Paris, Ile de France, France; 5 Hendaye Hospital, Route Corniche, 64700 Hendaye, Assistance Publique-Hôpitaux de Paris, France; 6 Committee for Studies, Education and Care for People With Multiple Disabilities (Comité d’Études, d’Éducation Et de Soins Auprès Des Personnes Polyhandicapées, CESAP), 62 Rue de La Glacière, 75013 Paris, France; 7 Service de Polyhandicap Pédiatrique, Roche Guyon Hospital, Assistance Publique Hôpitaux de Paris, 1 Rue Justinien Blazy 95780, La Roche-Guyon, France; 8 Hospital Fédération Des Hôpitaux de Polyhandicap Et Multihandicap, San Salvadour Hospital, Assistance Publique Hôpitaux de Paris, 4312 Rte de L’Almanarre, 83400 Hyères, France

**Keywords:** Care management, PIMD, Polyhandicap, Adequacy, Health consumption

## Abstract

**Background:**

The care organization of persons with profound intellectual and multiple disabilities (PIMD) varies by country according to the health care system. This study used a large sample of French individuals with severe PIMD/polyhandicap to assess: 1) the adequacy of care setting over a 5-year period and 2) health care consumption.

**Methods:**

The longitudinal study used data from the French EVALuation PoLyHandicap (EVAL-PLH) cohort of persons with severe PIMD/polyhandicap who were receiving managed in specialized care centres and residential facilities. Two assessments were performed: wave 1 (T1) in 2015–2016 and wave 2 (T2) in 2020–2021. The inclusion criteria were as follows: age > 3 years at the time of inclusion; age at onset of cerebral lesion younger than 3 years old; and severe PIMD. The adequacy of the care setting was based on the following: i) objective indicators, i.e., adequacy for age and adequacy for health status severity; ii) subjective indicators, i.e., self-perception of the referring physician about medical care adequacy and educational care adequacy. Health care consumption was assessed based on medical and paramedical care.

**Results:**

Among the 492 persons assessed at the 2 times, 50% of individuals at T1 and 46% of individuals at T2 were in an inadequate care setting based on age and severity. Regarding global subjective inadequacy, the combination of medical adequacy and educational adequacy, 7% of individuals at T1 and 13% of individuals at T2 were in an inadequate care setting. At T2, a majority of individuals were undermonitored by medical care providers (general practitioners, physical medicine rehabilitation physicians, neurologists, orthopaedists, etc.). Important gaps were found between performed and prescribed sessions of various paramedical care (physiotherapy, occupational therapy, psychomotor therapy, etc.).

**Conclusions:**

This study revealed key elements of inadequate care management for persons with severe PIMD/polyhandicap in France. Based on these important findings, healthcare workers, familial caregivers, patients experts, and health decision-makers should develop appropriate care organizations to optimize the global care management of these individuals.

**Trial registration:**

NCT02400528, registered 27/03/2015.

**Supplementary Information:**

The online version contains supplementary material available at 10.1186/s12913-024-10552-9.

## Introduction

People with profound intellectual and multiple disabilities (PIMD) are a heterogeneous group of individuals [1] characterized by a combination of profound intellectual disability and serious motor deficit, resulting in extreme restriction of autonomy and communication. When the disorder affects an immature brain, the term of polyhandicap is used. Polyhandicap, as a subgroup of PIMD, includes the most severe cases due to the precocity of the brain affection. Recently, the Ithaca European Reference Network for congenital malformations and rare intellectual disabilities has agreed on the term PIMD/Polyhandicap [2]. While the individuals may present varying disorders and comorbidities, they all depend on human and technical assistance, and they all need permanent health and educational support [3]. Specific care and services are necessary for people with PIMD/Polyhandicap including physical medicine rehabilitation and other medical specialties, as well as physiotherapy, occupational therapy, psychomotor therapy, and speech therapy. As these individuals are at greater risk of experiencing severe complications (including epilepsy and respiratory infections) [4], intensive care medical services should be available at all times to prevent deterioration.

The healthcare organization of persons with PIMD/Polyhandicap differs by country, depending both on the specificities of the associated health care system and the related societal views [5, 6]. In contrast to other European countries with extensive deinstitutionalization processes, the French system relies on institutional settings aiming to offer a graduated response adapted to the individuals health status. The French health system allows these patients to benefit from two main care management modalities: specialized rehabilitation centres and residential facilities [7, 8]. Specialized rehabilitation centres offer a high level of medical and paramedical physical rehabilitation and a high level of preventive care for inpatients for a theoretically limited duration. Residential facilities offer a high level of psychosocial education and a lower level of medical care. Within these two modalities, there are units dedicated to adult populations (in- or outpatients over 18 years old) and units dedicated to paediatric populations (in- or outpatients units under 18 years old). Some persons (children and adults) are cared for at home care; in this case, the family benefits from help with nursing and medical care.

This offer is supposed to optimize the care management of persons with severe polyhandicap according to their specific needs in terms of age, health severity, and medical and educational care. Few data describing the adequacy of care setting are available. The first related French study [9], which was performed in 2015, provided important information from a large sample of persons with severe polyhandicap. From the perspective of the population studied therein (child or adult population), this study showed that approximately 10% of the individuals were not care managed in a structure adapted to their age (i.e., persons under than 18 years old who were cared for in a unit dedicated to adults and persons over 18 years old who were cared for in a unit dedicated to children). Almost half of the individuals were not receiving care in an appropriate structure from the perspective of health severity (i.e., some people with the most severe health status were receiving care in residential facilities, and some people with less severe health status were receiving care in specialized rehabilitation centres). If we can assume that being in the adequate structure (age, health severity) better meets the true needs of the persons, health policy decisions to close or open structures could be based on this valuable data.

To improve the global adequacy of care settings, providing better knowledge of the care consumption of these individuals would strongly improve the health policies and resource allocation. To date, there is no robust inventory of the use of medical and paramedical care among individuals, thereby resulting in suboptimal health care.

In this paper, we used a large sample of French persons with severe polyhandicap to examine: 1) the evolution of the adequacy of care setting over a 5-year period (from 2015–16 to 2020–21) and 2) the health (medical and paramedical) care services used by this population. The results were provided for the whole sample and according to the following subgroups: i) residential facilities and specialized rehabilitation centres and ii) children and adults.

## Methods

### Design and settings

The study used data from the French cohort (EVALuation PoLyHandicap EVAL-PLH) of individuals with severe polyhandicap. Details of the study protocol of the cohort were published elsewhere [10]. Data were collected at 2 points: the first wave (Time 1, T1) was collected in 2015 and 2016, and the second wave (Time 2, T2) was collected in 2020 and 2021. Persons with severe PIMD who were cared for in 4 specialized rehabilitation centres (SRC) and 9 residential facilities (RFs) were eligible.

### Ethics

Regulatory monitoring was performed in accordance with French law that requires the approval of the French ethics committee (Comité de Protection des Personnes Sud Méditerranée V, 20/10/2014, reference number 2014-A00953-44). A written consent form was collected for each participant (from the legal representative). Clinical trial number: NCT02400528 (registered 27/03/2015).

### Selection criteria

The inclusion criteria were as follows: age over 3 years at the time of inclusion; polyhandicap defined by: i) a cerebral lesion leading to a combination of motor deficiency (tetraparesia, hemiparesis, paraparesis, extrapyramidal syndrome, cerebellar syndrome, and/or neuromuscular problems), profound intellectual impairment (intelligence quotient IQ < 40) associated with everyday life dependence (Functional Independency Measure FIM < 55), and restricted mobility (Gross Motor Function Classification System GMFCS III, IV, and V); ii) age at onset of the cerebral lesion younger than 3 years old; and iii) usual care setting in specialized rehabilitation centres or residential facilities.

### Samples

The evolution of adequacy was studied from the sample of persons who were assessed at the 2 evaluation times, 2015–2016 and 2020–2021 (sample 1) and the sample of persons assessed at T2 (sample 2). The health care consumption was studied from the sample of persons assessed at T2 (sample 2).

### Data collection

For each person, the following data were available in the case report form: sex, age, aetiology lesion time (antenatal, perinatal, and postnatal), aetiology nature (nonprogressive and progressive), mobility (GMFCS), independency (FIM score), profound intellectual impairment (IQ), age classes at assessment (two classes: < 18 or > = 18 years), usual care setting (specialized rehabilitation centres or residential facilities), severity of health status. Severe health status was defined by motor handicap (including paraparesis, tetraparesia, extrapyramidal syndrome, or severe general hypotonia), an IQ < 25, a FIM score ≤ 20, and a GMFCS IV and V. Otherwise, persons were considered to have less severe health status. A specific monitoring was performed by the administrative coordinator to identify people who moved or died over time.

### Definition of adequacy of care setting

The adequacy of the care setting was based on objective adequacy and subjective adequacy defined as follows:

–Objective adequacy:

Adequacy for age: Adequacy was defined as a person under 18 years old who was cared for in a unit dedicated to children or by a person over 18 years old who was cared for in a unit dedicated to adults. Inadequacy was defined as either of 2 situations: a person under 18 years old who was cared for in a unit dedicated to adults or by a person over 18 years old who was cared for in a unit dedicated to children.

Adequacy for health status severity: Adequacy was defined as a person with a severe health status who was cared for in a specialized rehabilitation centre or by a person with less severe health status who was cared for in residential facilities. Inadequacy was defined as either of 2 situations: a person with a severe health status who was cared for in residential facilities or by a person with less severe health status who was cared for in a specialized rehabilitation centre.

Objective adequacy was a combination of age adequacy and severity adequacy. Objective adequacy was defined as age adequacy and severity adequacy. Inadequacy was defined as any other case.

–Subjective adequacy:

Adequacy for medical care: Adequacy was defined based on the self-perception of the referring physician of the individual about medical care provided in the setting.

Adequacy for educational care: Adequacy was defined based on the self-perception of the referring physician of the individual about educational care provided in the setting.

Subjective adequacy was a combination of medical adequacy and educational adequacy. Subjective adequacy was defined as adequate medical care and adequate educational care. Inadequacy was defined as any other case.

### Definition of health care consumption

Health care consumption was assessed using the following indicators:

- Consumption of medical care: The number of medical consultations during the last year before the assessment were recorded for general practitioner, physical medicine rehabilitation, neurologist (epilepsia, sleep disorders), dentist, orthopaedist (scoliosis, hip luxation), gastrologist, pneumologist, ophthalmologist, paediatrician (for children), and gynaecologist (for girls-women).
- Consumption of hospitalization stays: During the last year before the assessment, the number of planned conventional hospitalizations, unplanned conventional hospitalizations, and intensive care unit stays were recorded.
- Occurrence of decompensation: The number of decompensation episodes leading to admission to an acute care unit during the last year before the assessment was recorded.
- Consumption of paramedical care: For each item, the monthly prescribed sessions and the gap between the number of prescribed and performed sessions were recorded: physiotherapy, occupational therapy, psychomotor therapy, speech therapy, orthoptist, psychologist, special educator, and dietitian. The gap of prescribed/performed sessions was defined by the number of prescribed sessions minus number of performed sessions (D);3 categories were used: no gap (D < 3 sessions), minor gap (3 < = D < = 10), large gap (D > 10).

## Statistical analysis

The proportions of each indicator of adequacy were provided for the 2 samples (sample 1, *N* = 492 paired subjects, and sample 2, *N* = 619). The description of medical and paramedical consumption was provided for sample 2 (*N* = 619): i) all the sample; ii) according to the usual care setting (specialized rehabilitation centres or residential facilities), iii) according to the age classes (< or > = 18 years). Quantitative data are expressed as the medians and interquartile ranges (IQR), and qualitative data are expressed as numbers and percentages. The analyses were performed using SPSS software (IBM SPSS PASW Statistics Inc., Chicago, IL, USA).

## Results

### Populations

The main characteristics of sample 1 (persons assessed at the 2 evaluation times, *N* = 492) and sample 2 (persons assessed at T2, *N* = 619) are detailed in additional file 1 (see Additional Table 1. Sample characteristics).

**Table 1:** Medical care during the last year before assessment (*N* = 619)

		**All**		**SRC**		**RF**		**Children (< 18y)**		**Adults (> = 18y)**	
		***N* = 619**		***N* = 297**		***N* = 322**		***N* = 199**		***N* = 420**	
**Number of medical consultations**		**N**	**%**	**N**	**%**	**N**	**%**	**N**	**%**	**N**	**%**
**General practitioner**	none	313	53.2	141	47.6	172	58.9	105	59	208	50.7
	1 to 5	174	29.6	83	28.1	91	31.1	58	32.5	116	30.7
	> 5	101	17.2	72	24.3	29	10	15	8.5	86	18.5
	Missing	31		1		30		21		10	
**Physical medicine rehabilitation physician**	none	339	59.4	164	55.4	175	63.6	95	54	244	61.8
	1	127	22.2	53	17.9	74	26.9	44	25	83	21
	> 1	105	18.4	79	26.7	26	9.4	37	21	68	17.2
	Missing	48		1		47		23		25	
**Neurologist**	none	415	73.2	220	74.3	195	72	94	53.7	321	81.9
	1	104	18.3	56	18.9	48	17.7	55	31.4	49	12.5
	> 1	48	8.5	20	6.8	28	10.3	26	13.9	22	5.6
	Missing	52		1		51		24		28	
**Orthopaedist**	none	431	77	212	72.1	219	82.3	107	62.6	324	83.3
	1	81	14.5	48	16.3	33	12.4	47	27.5	34	8.7
	> 1	48	8.6	34	11.5	14	5.3	17	9.9	31	7.9
	Missing	59		3		56		28		31	
**Dentist**	none	250	44.4	41	13.9	209	78.3	91	55.5	159	39.8
	1	146	25.9	107	36.1	39	14.6	29	17.7	117	29.3
	> 1	167	29.7	148	50	19	7.1	44	26.8	123	30.8
	Missing	56		1		55		35		21	
**Gastrologist**	none	490	88.6	267	90.2	223	86.8	128	78.5	362	92.8
**Pneumologist**	none	503	91	266	89.6	237	92.6	141	86	362	93.1
**Ophtalmologist**	none	512	92.6	270	91.2	242	94.2	133	82.1	379	96.9
**Paediatrician (only for individuals < 18y)**	none	NA		68/78	87.2	66/88	75%	134/166	80.7	NA	
**Gynaecologist (only for girls-women)**	none	201/249	80.7	92/136	67.6	109/113	96.4	62/65	95.4	139/184	75.5

*SRC* Specialized rehabilitation centre, *RF* Residential facilities, *N* numbers, % Percent, *NA* Not applicable

### Objective adequacy of care settings

**Fig. 1:**
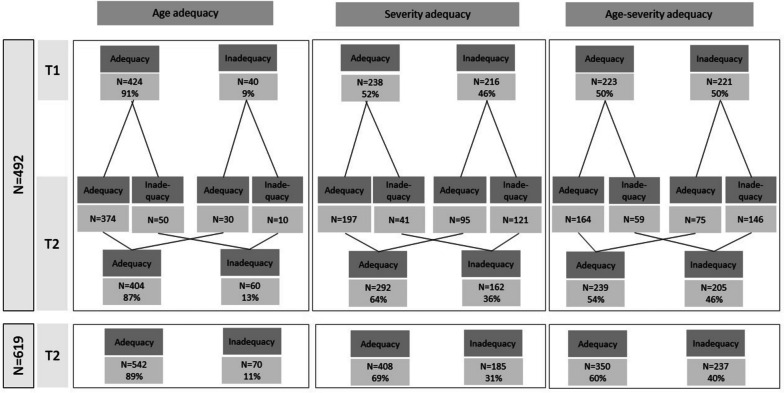
Evolution of objective adequacy of care setting over the 5-year period. Adequacy for age: Adequacy was defined as an individual under 18 years old who was cared for in a unit dedicated to children or by an individual over 18 years old who was cared for in a unit dedicated to adults. Adequacy for health status severity: Adequacy was defined by an individual with a severe health status who was cared for in a specialized rehabilitation centre or by a patient with less severe health status who was cared for in residential facilities. Combination of age adequacy and severity adequacy: Adequacy was defined as age adequacy and severity adequacy. T1: 2015–2016 assessment; T2: 2020–2021 assessment

Among the 492 persons (sample 1), according to the previous definition of age adequacy, 9% and 13% of them were in an inadequate care setting at T1 and T2, respectively. Of the 40 inadequate individuals at T1, 30 were classified as adequate and 10 were classified as inadequate at T2. In parallel, 50 individuals of the 424 individuals in an adequate care setting at T1 were in an inadequate situation at T2. The inadequacy mostly corresponded to adult care managed in a paediatric unit. From the perspective of severity adequacy, 46% of individuals at T1 and 36% at T2 were classified as inadequate. At T1, 54% of individuals in inadequate situations were persons in non-severe health status who were care for in specialized rehabilitation centres that were intended to provide intensive medical care, and 46% were persons with severe health status who were cared for in residential facilities. The global objective adequacy (combination of age adequacy and severity adequacy) showed a quite similar proportion of inadequate situation between T1 and T2: 50% and 46%, respectively. Among the entire sample assessed at T2 (sample 2, *N* = 619), the proportions of persons classified as inadequate due to age, severity, and combination were 11%, 31%, and 40%, respectively. All the details are provided in Fig. 1.

### Subjective adequacy of care settings

**Fig. 2:**
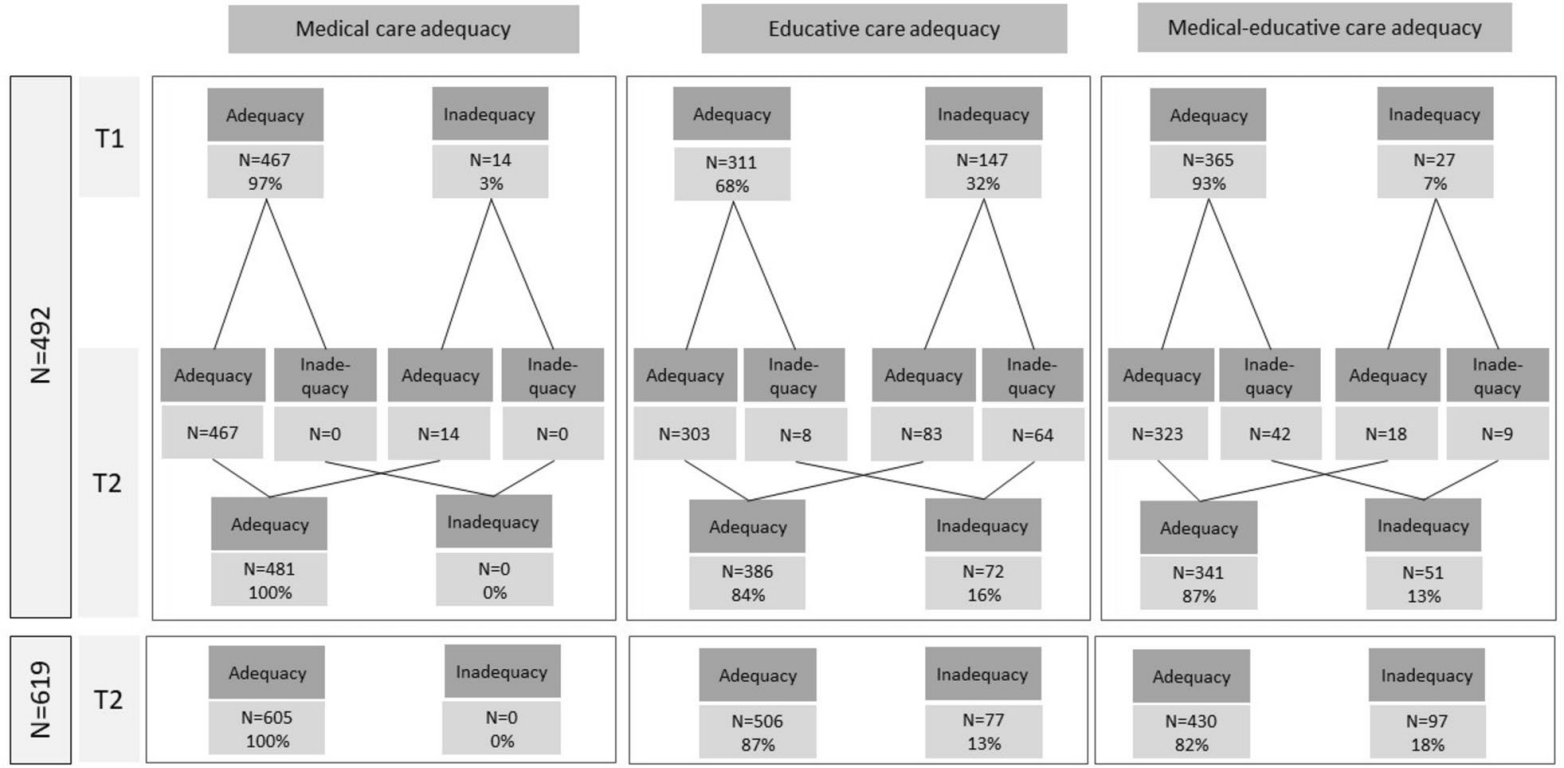
Evolution of subjective adequacy of care setting over the 5-year period. Adequacy for medical care: Adequacy was defined based on the self-perception of the referring physician of the individual about medical care provided in the setting. Adequacy for educational care: Adequacy was defined based on the self-perception of the referring physician of the individual about educational care provided in the setting. Combination of medical adequacy and educational adequacy: Adequacy was defined as adequate medical care and adequate educational care. T1: 2015–2016 assessment; T2: 2020–2021 assessment

Among sample 1 (*N* = 492), the physicians’ perception of medical care inadequacy was low at T1 (3%) and null at T2. The physicians’ perception of educational care inadequacy was higher: 32% at T1 and 16% at T2. The global subjective inadequacy, as the combination of medical adequacy and educational adequacy, was 7% and 13%, at T1 and T2, respectively. Among sample 2 (*N* = 619), inadequacies for medical care, educational care, and combination were null, 13%, and 18%, respectively. All the details are provided in Fig. 2.

### Health care consumption

Among the 619 persons assessed at T2 (sample 2), more than half had not been examined by a general practitioner during the last year before the assessment. This proportion was more important for the persons cared for in residential facilities (59%) and for the children (59%). A high proportion (59%) of persons were not examined by physical medicine rehabilitation (64% when considering persons cared for in residential facilities and 62% when considering adults). The proportions of persons who had been seen at least one time in the last year by a neurologist and an orthopaedist were 27% and 23%, respectively. The proportion of persons who had not a dentist consultation was 44%; this proportion was much lower for persons in specialized rehabilitation centres (14%), and remained high for children (56%). During the last year, more than 80% of the children were not seen by a paediatrician and more than 75% of the women were not seen by a gynaecologist. All the details are provided in Table 1.

During the last year before assessment, for the whole sample, planned and unplanned hospitalization episodes were reported for 15% and 11%, respectively; persons in residential facilities (17 and 18%, respectively) or children (32 and 20%, respectively) were the most affected. Three percent of the sample was admitted to the intensive care unit. A quarter of the sample and a third of persons cared for in specialized rehabilitation centres reported at least one decompensation episode. The main decompensation cause was pneumopathy (from 38 to 81% of cases according to the groups). All the details are provided in Table 2.

**Table 2:** Hospitalization stays during the last year before assessment (*N* = 619)

		**All**		**SRC**		**RF**		**Children (< 18y)**		**Adults (> = 18y)**	
		***N* = 619**		***N* = 297**		***N* = 322**		***N* = 199**		***N* = 420**	
		**N**	**%**	**N**	**%**	**N**	**%**	**N**	**%**	**N**	**%**
**Planned hospitalization stays**	none	461	84.7	252	86	209	83.3	119	69.2	342	91.9
	> = 1	83	15.3	41	14	42	16.7	53	31.8	30	8.1
	number of days, m [IQR]	2[1–6]		1[1–4]		2[1–9]		2[1–7]		1[1–3]	
	MD	75		4		71		27		48	
**Unplanned hospitalization stays**	none	485	89.3	279	95.5	206	82.1	136	80	349	93.6
	> = 1	58	10.7	13	4.5	45	17.9	34	20	24	6.3
	number of days, m [IQR]	5[2–9]		4[1–8]		5[2–14]		5[2–14]		5[1–8]	
	MD	76		5		71		29		47	
**ICU stays**	none	523	96.7	284	97.3	239	96	156	92.9	367	98.4
	> = 1	18	3.3	8	2.7	10	4	12	7.1	6	1.6
	number of days, m [IQR]	3[1–10]		4[1–6]		3[0–23]		3[0–9]		4[3–31]	
	MD	78		5		73		31		47	
**Decompensation episode**	none	405	74.7	203	69.5	202	80.8	125	74	280	75.1
	> = 1	137	25.3	89	31.5	48	19.2	44	26	93	24.9
	MD	77		5		72		30		47	
**Main decompensation cause**	pneumopathy	63	46	45	50.6	18	37.5	25	56.8	38	40.9
	epilepsy	11	8	4	4.5	7	14.6	6	13.6	5	5.4
	others	63	46	40	44.9	23	47.9	13	29.5	50	53.8

*SRC* Specialized rehabilitation centre, *RF* Residential facilities, *N* Numbers, % Percents, *MD* Missing data, *ICU* Intensive care unit, m [IQR] median [interquartile range]

During the last month before assessment, no sessions of physiotherapist were prescribed for 25% of the persons. Almost half of the persons had no prescribed sessions of occupational therapy and 56% had no prescribed sessions of psychomotor therapy. All these proportions were higher for the persons cared for in specialized rehabilitation centres and for the adults and lower for the persons cared for in residential facilities and for the children. Speech therapy was prescribed to 10 to 20% of the persons, depending on the groups. Orthoptics was rarely prescribed, most often for children (11%). Psychology sessions were prescribed for 47% of the sample, 25% of the persons cared for in specialized rehabilitation centres, 70% of the persons cared for in residential facilities, 60% of the children, and 42% of the adults. No special educator sessions were prescribed for one third of the persons. Dietitians were prescribed for 53% of the sample, ranging from 30% in residential facilities to 70% in specialized rehabilitation centres. All the details are provided in Table 3.

**Table 3:** Paramedical care during the last month before assessment (*N* = 619)

		**All**		**SRC**		**RF**		**Children (< 18y)**		**Adults (> = 18y)**	
		***N* = 619**		***N* = 297**		***N* = 322**		***N* = 199**		***N* = 420**	
		**N**	**%**	**N**	**%**	**N**	**%**	**N**	**%**	**N**	**%**
Prescribed physiotherapy sessions	no	149	25.3	91	30.6	58	19.9	8	4.3	141	35.1
	yes (at least one)	440	74.7	206	69.4	234	80.1	179	95.7	261	64.9
Number of sessions performed	m [IQR]	8 [4-16]		16 [8-30]		8 [4-12]		12 [6-30]		8 [4-12]	
Prescribed occupational therapy sessions	no	291	49.2	202	68	89	30.2	61	33.5	230	56.1
	yes (at least one)	301	50.8	95	32	206	69.8	121	66.5	180	43.9
Number of sessions performed	m [IQR]	4 [2–6]		2 [2–2]		4 [2–8]		2 [2–4]		4 [2–8]	
Prescribed psychomotor therapy sessions	no	323	56	188	63.3	135	48.2	49	27.4	274	68.8
	yes (at least one)	254	44	109	36.7	145	51.8	130	72.6	124	31.2
Number of sessions performed	m [IQR]	4 [2–4]		2 [2–4]		4 [4–4]		4 [2–4]		4 [2–4]	
Prescribed speech therapy sessions	no	516	91.8	292	98.3	224	84.5	137	80.1	379	96.9
	yes (at least one)	46	8.2	5	1.7	41	15.5	34	19.9	12	3.1
Number of sessions performed	m [IQR]	4 [4–4]		2 [2–4]		4 [4–4]		4 [4–4]		4 [4–4]	
Prescribed orthoptics sessions	no	530	96.2	281	94.6	249	98	143	88.8	387	99.2
	yes (at least one)	21	3.8	16	5.4	5	2	18	11.2	3	0.8
Number of sessions performed		2 [2–2]		2 [1, 2]		4 [3–6]		2 [2–2]		4 [4–4]	
Prescribed psychology sessions	no	310	52.6	224	75.4	86	29.5	72	40.2	238	58
	yes (at least one)	279	47.4	73	24.6	206	70.5	107	59.8	172	42
Number of sessions performed		4 [1–4]		1 [1–1]		4 [4–4]		2 [1–4]		4 [4–4]	
Prescribed special educator sessions	no	189	33.7	97	32.8	92	34.7	56	33.7	133	33.7
	yes (at least one)	372	66.3	199	67.2	173	65.3	110	66.3	262	66.3
Number of sessions performed	m [IQR]	8 [4-30]		4 [2-4]		30 [20-30]		2 [0–4]		4 [0–20]	
Prescribed dietary sessions	no	257	46.6	80	26.9	177	69.7	73	45.3	184	47.2
	yes (at least one)	294	53.4	217	73.1	77	30.3	88	54.7	206	52.8
Number of sessions performed		1 [1–1]		1 [1–1]		1 [1–1]		1 [1–1]		1 [1–1]	

*SRC* Specialized rehabilitation centre, *RF* Residential facilities, *N* Numbers, % Percent, *MD* Missing data, *ICU* Intensive care unit, m [IQR] median [interquartile range]

**Fig. 3:**
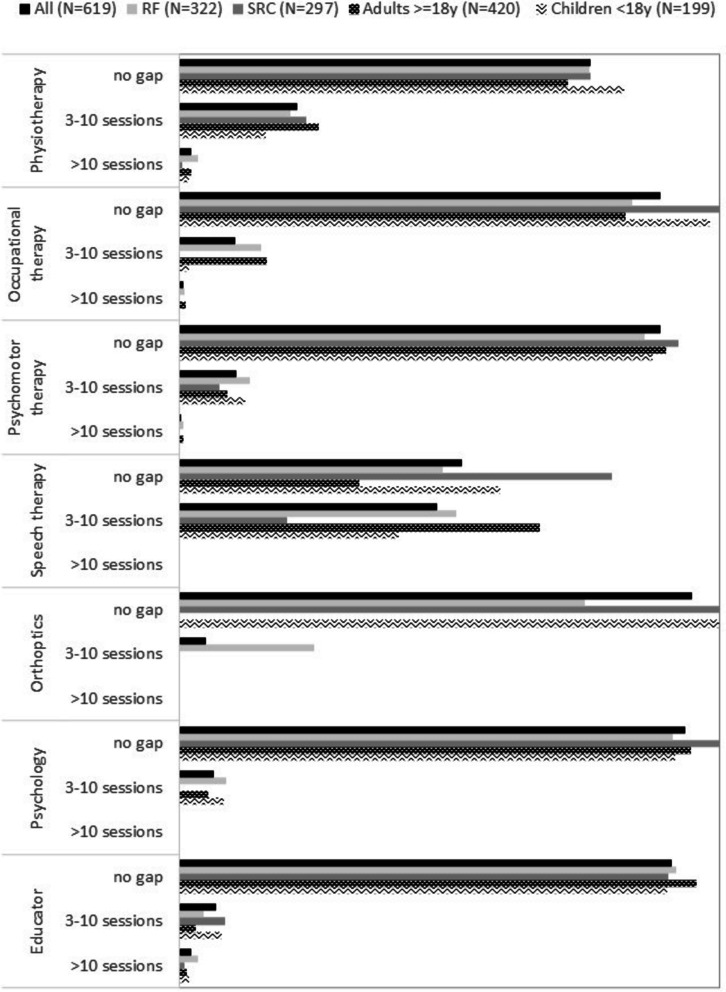
Gap between prescribed and performed paramedical sessions (*N* = 619). Gap prescribed/performed sessions: number of prescribed sessions minus number of performed sessions (D); no gap: D < 3, gap (3–10 sessions):3 < = D < = 10; large gap: D > 10. SRC specialized rehabilitation centre; RF residential facilities

The number of prescribed sessions differed from the number of performed sessions. The highest gap was for speech therapy, with almost half of the prescribed sessions that were not performed. Almost one-quarter of the prescribed sessions of physiotherapy were not performed. Eleven percent of prescribed occupational therapy sessions and prescribed psychomotor sessions were not performed. In general, the gap was higher in residential facilities than in specialized rehabilitation centres, and higher in adults than in children. All the details are provided in Fig. 3.

## Discussion

To our knowledge, this study is the first to describe the evolution of important indicators of care management adequacy for people with severe PIMD in France over a 5-year period.

The first important finding was that objective adequacy, defined as a combination of age adequacy (i.e., a child cared for in a child unit or an adult who was cared for in an adult unit) and severity adequacy (i.e., a severe person cared for in a specialized rehabilitation centres or a less severe person cared for in residential facilities), has improved over time (46% and 50% in 2015–2016 and 2020–2021, respectively). However, the rates of inadequacy are still high. Part of this inadequacy concerned persons who were not care managed in an appropriate care setting according to their age. Adults care managed in units dedicated for children represented 95% of these cases. Another part of the objective inadequacy concerned people who were not care managed in an appropriate care setting according to their health severity. This inadequacy may have consequences. The French health decision-making agencies can exhort the institutions to position the patients according to the legal age (18 years) or the severity. And, in case of non-compliance, beds or units can be suppressed.

Based on these 2 observations, several hypotheses could be suggested. First, the inadequacy could be due to a lack of places (in particular, a lack of places in units dedicated to adult persons) and to a heterogeneous offer of care settings on the French territory, thereby resulting in underresourced areas. Due to the scarcity of specialized centres, the person may be moved far from their family’s place of residence. Families and support teams may choose a less appropriate (in terms of age and/or severity) but nearby care setting rather than a more appropriate care setting that is farther away. These findings could also be relevant for health decision-makers in the planning of future health organizations for persons with severe PIMD. Decision-makers should consider increasing the number of beds dedicated to adults and thinking about a wider and more homogeneous geographical offer of places. Second, the objective inadequacy could be due to a reluctance from the families. Indeed, previous studies showed that parents could be worried by the idea that their child is going to change units [11]. A change of the familiar environment (change of the care team, change of care structure) could be at high risk of decompensation for the person [12], sometimes leading to the concept of “failure to cope” [13]. In addition to the families’ reluctance, it is not sensible to think that the care team, knowing and taking care of the persons for a long time, tied to the persons [14], could be in a passive position regarding the admission of their patients to another centre. Improving the transition between paediatric and adult health care is not only promoted for persons with PIMD but also widely debated in other fields, such as various chronic diseases [15, 16]. A cultural shift in staff attitudes and effective transition programs are required [17]. Currently, to change representations and beliefs, it is essential to better connect adult and paediatric care settings [18], medicosocial and medical structures, and families and health care teams [19].

The rate of subjective adequacy, defined by a combination of medical adequacy and educational adequacy perceived by the referring physician, was better than the rate of the objective adequacy but decreased over time from 93% (2015–2016) to 87% (2020–2021). Global inadequacy was mainly due to self-perceived inadequacy for educational care, while adequacy for medical care was at the maximal level. This finding is unsurprising. It is now well-recognized that educational care remains essential for PIMD persons [20, 21]. Behaviourial disorders, teeth grinding, self-injury, and autistic-like traits should be partially controlled [22]. Many care settings lack resources to enhance educational care. The gap between performed and prescribed sessions of various paramedical care, including special educators, enhances this result. Health care teams consider that educational care, as well as care-like care, such as physiotherapy, occupational therapy, and psychomotor therapy, is essential in the global care management of persons with PIMD. However, care settings cannot provide these services due to insufficient resources. In the future, it would be important to think about care settings that offering mixed care, including more balanced medical and educative care. It would also be relevant to assess the adequacy perceived by familial caregivers: they are also concerned and can provide a complementary point of view.

Finally, this study provided detailed information on the health consumption of PIMD/polyhandicap persons. While various specific medical consultations seem essential to organize preventive and curative treatments, we found that many persons were insufficiently monitored by general practitioners and physical medicine rehabilitation physicians. Specific needs of persons with PIMD require frequent monitoring: optimization of epilepsy treatment [23], detection of scoliosis or other deformations, diagnosis of gastroesophageal reflux and maintenance of gastrostomy [24], and pain evaluation [25, 26]. Experimented paediatricians and gynaecologists may be of high interest in the medical follow-up of the target persons (children and women, respectively). Oral health maintenance must also be considered [27]. Therefore, suboptimal management improves the risk of health deterioration or decompensation episodes and consequently the risk of unplanned admissions in conventional medical settings or, more troublesomely, intensive care admission. Administrative and medical staff of these settings, both residential facilities and specialized centres, regularly request more financial resources to be able to offer more appropriate care. Future health care decisions could be based on these robust findings.

Some limitations should be discussed. First, the data collection having been carried out during the year 2020–2021, we can hypothesize that the organization of care could have been significantly modified by the SARS-CoV-2 pandemic. Work absenteeism and safety guidelines, such as limiting human contact and postponing non-urgent care, may have reduced access to care, including medical consultations and paramedical care. Previous studies described the impact of the pandemic on the care organizations in France [28, 29]. The absence of similar data from the 1st wave deprives us of reference. The third wave of the cohort will allow to better explore this hypothesis. Second, we only collect healthcare consumption defined by medical consultations, hospitalizations, and paramedical care. However, future medico-economic studies should be considered on the basis of an exhaustive identification of the costs of care, including direct costs (drugs, medical devices and wheelchairs) and indirect costs.

## Conclusion

This study revealed some key elements of the inadequate care management for severe PIMD/polyhandicap in France. Based on these important findings, healthcare workers, familial caregivers, patients experts, and health decision-makers should develop appropriate care organizations to optimize the global care management of these individuals.

### Supplementary Information

**Additional file 1. **

## Data Availability

The datasets used and/or analysed during the current study are available from the corresponding author on reasonable request.

## Abbreviations

EVAL-PLH: EVALuation PoLyHandicap
FIM: Functional Independency Measure
GMFCS: Gross Motor Function Classification System
IQ: Intelligence quotient
PIMD: Profound intellectual and multiple disabilities
RF: Residential facilities
SRC: Specialized rehabilitation centres
