# Supplementary material for: Health care management adequacy among French persons with severe profound intellectual and multiple disabilities: a longitudinal study
Source: BMC Health Serv Res. 2024 Jan 18;24:99. doi: 10.1186/s12913-024-10552-9 (PMC10795329; doi:10.1186/s12913-024-10552-9)
Supplement: Supplementary file 1 — Additional file 1. [file 12913_2024_10552_MOESM1_ESM.docx]

**Additional table 1. Sample characteristics**

|  |  |  | **Sample 1**  **T1 and T2** | | **Sample 2**  **T2** | |
| --- | --- | --- | --- | --- | --- | --- |
|  |  |  | **N=492** | **MD%** | **N=619** | **MD%** |
| Sex | Boys/Men | N (%) | 262 (53.3) | 0 | 332 (53.6) | 0 |
|  | Girls/Women | N (%) | 230 (46.7) |  | 287 (46.4) |  |
| Age at the time of inclusion |  | M (SD) | 28.2 (16.5) | 0 | 29.9 (17.0) | 0 |
|  |  | Med [IQR] | 24 [14-44] |  | 15 [15-45] |  |
| Aetiology lesion time | Antenatal | N (%) | 195 (49.6) | 20.1 | 257 (51.3) | 19.1 |
|  | Perinatal | N (%) | 102 (26.0) |  | 127 (25.3) |  |
|  | Postnatal | N (%) | 58 (14.8) |  | 75 (15.0) |  |
|  | Non-classifiable | N (%) | 38 (9.7) |  | 42 (8.4) |  |
| Aetiology nature | Nonprogressive | N (%) | 322 (74.5) | 12.2 | 397 (73.5) | 12.8 |
|  | Progressive | N (%) | 81 (18.8) |  | 104 (19.3) |  |
|  | Nonclassifiable | N (%) | 29 (6.7) |  | 39 (7.2) |  |
| Mobility (GMFCS) | III | N (%) | 47 (9.6) | <1 | 69 (11.2) | <1 |
|  | IV | N (%) | 109 (22.3) |  | 49 (8.0) |  |
|  | V | N (%) | 332 (68.0) |  | 497 (80.8) |  |
| Functional independency | (FIM score) | Med [IQR] | 20 [18-25] | <1.0 | 21 [18-27] | <1 |
| Profound intellectual impairment | Yes | N (%) | 416 (84.9) | <1 | 588 (97.5) | 2.6 |
|  | No | N (%) | 74 (15.1) |  | 15 (2.5) |  |

T1: 2015-16 evaluation ; T2: 2020-21 evaluation.

N (%): number (percent); M (SD): mean (standard deviation); Med [IQR]: median [interquartile range].

Missing: missing data

GMFCS: Gross Motor Function Classification System.

FIM: Functional Independency Measure.

Profound intellectual impairment: Intelligence quotient <25 or non-evaluable.
